# *Porphyromonas gingivalis* regulates atherosclerosis through an immune pathway

**DOI:** 10.3389/fimmu.2023.1103592

**Published:** 2023-03-14

**Authors:** Qijun Ruan, Peng Guan, Weijuan Qi, Jiatong Li, Mengying Xi, Limin Xiao, Sulan Zhong, Dandan Ma, Jia Ni

**Affiliations:** ^1^ Department of Periodontics, Stomatological Hospital, School of Stomatology, Southern Medical University, Guangzhou, China; ^2^ Department of Endodontics, Stomatological Hospital, School of Stomatology, Southern Medical University, Guangzhou, China

**Keywords:** *porphyromonas gingivalis*, atherosclerosis, immune escape, blood circulation, lymphatic circulation

## Abstract

Atherosclerosis (AS) is a chronic inflammatory disease, involving a pathological process of endothelial dysfunction, lipid deposition, plaque rupture, and arterial occlusion, and is one of the leading causes of death in the world population. The progression of AS is closely associated with several inflammatory diseases, among which periodontitis has been shown to increase the risk of AS. *Porphyromonas gingivalis* (*P. gingivalis*), presenting in large numbers in subgingival plaque biofilms, is the “dominant flora” in periodontitis, and its multiple virulence factors are important in stimulating host immunity. Therefore, it is significant to elucidate the potential mechanism and association between *P. gingivalis* and AS to prevent and treat AS. By summarizing the existing studies, we found that *P. gingivalis* promotes the progression of AS through multiple immune pathways. *P. gingivalis* can escape host immune clearance and, in various forms, circulate with blood and lymph and colonize arterial vessel walls, directly inducing local inflammation in blood vessels. It also induces the production of systemic inflammatory mediators and autoimmune antibodies, disrupts the serum lipid profile, and thus promotes the progression of AS. In this paper, we summarize the recent evidence (including clinical studies and animal studies) on the correlation between *P. gingivalis* and AS, and describe the specific immune mechanisms by which *P. gingivalis* promotes AS progression from three aspects (immune escape, blood circulation, and lymphatic circulation), providing new insights into the prevention and treatment of AS by suppressing periodontal pathogenic bacteria.

## 1 Introduction

*P. gingivalis*, a Gram-negative anaerobic bacterium, is the “dominant flora” in periodontitis (1, 2). *P. gingivalis* can stimulate the host immune response through virulence factors, including its structural components (fimbriae, LPS, etc.) and secretory components (gingipains and OMVs) (3). *P. gingivalis* fimbriae can enhance the inflammatory response and evade host immune clearance (4). *P. gingivalis* LPS is an important virulence factor with lipid A as its immune core, triggering a signaling pathway closely related to lipid A variants (5). *P. gingivalis* gingipains are powerful protein hydrolysers that assist *P. gingivalis* to evade host immunity (6). *P. gingivalis* OMVs are outer membrane vesicles containing multiple virulence factors that can circulate widely throughout the body and access areas of tissue not accessible to whole bacteria, thereby triggering an inflammatory response (7). *P. gingivalis* and its virulence factors can promote the development of a variety of systemic diseases such as cardiovascular disease, diabetes, Alzheimer’s disease, etc (8).

AS is a chronic inflammatory pathological change occurring in the walls of medium to large arteries, characterized by immune cell infiltration and lipid deposition, and poses a serious threat to human life and health. Recent single-cell sequencing studies have revealed abundant heterogeneity of immune cells in the AS plaque environment. For example, three macrophage subpopulations were identified in human and mouse AS plaques, including inflammatory, resident-like (TREM2^hi^), and TREM2^hi^ macrophages. Among them, TREM2^hi^ macrophages are a foam-like, anti-inflammatory type of macrophages and exhibit an osteoclast-like gene expression profile that may be associated with plaque calcification (9, 10). Similar studies point to the presence of a cluster in mouse AS plaques that matches the core marker profile of B1 cells (CD43^high^B220^neg^CD11b^high^) but shows enriched TNF signaling and cell adhesion pathways that may be key cell types for promoting AS (11). Previously unknown clusters of naive T cells and ApoB^+^ T cells have also been found in plaques and are closely associated with vascular inflammation, but the exact mechanism is not yet clear (12). Another study found that vascular smooth muscle cells (VSMCs) phenotype-switching mechanisms play an important role in AS. During AS development, VSMCs can give rise to a novel intermediate cell with multidirectional differentiation potential, which can either differentiate into macrophage-like or fibrocartilage-like cells or revert to VSMCs (13). VSMCs and endothelial cells (ECs) in the core of human arterial AS lesions were found to drive cellular transdifferentiation through multiple genes, whereas VSMCs and ECs in the adjacent zone were involved in immune cell recruitment through C3 and MHC II molecules, respectively (14). More in-depth studies using single-cell sequencing pointed out that DHRS9 in macrophages is a key factor in AS formation (15), and CXCL3, GK, FPR1, and LST1 in monocytes are closely associated with plaque instability (16). The application of single-cell sequencing technology has deepened the understanding of cellular heterogeneity in AS lesions, and provided an important theoretical basis for further investigation of AS pathogenesis and the development of targeted therapeutic drugs. In addition, single-cell sequencing studies have the advantage of investigating the mechanisms of interaction between two or more cells in tissues, such as between immune cells and tissue cells, yet no relevant studies have been seen.

Existing researches show that *P. gingivalis* promotes AS progression through immune responses (17, 18). On the one hand, *P. gingivalis* evades host innate and adaptive immunity and, in various forms, circulates with blood and lymph and colonizes the arterial vessel wall, directly inducing local inflammation and lesions in blood vessels. On the other hand, after evading host immunity, *P. gingivalis* persistently stimulates the host immune response, induces systemic inflammatory mediators and autoimmune antibody production, disorders of lipid levels, and promotes AS progression. In this paper, we review the recent evidence on *P. gingivalis* promoting AS and the related immune response mechanisms to elucidate the potential mechanism of action and association between *P. gingivalis* and AS, and provide theoretical guidance for further in-depth studies.

## 2 Evidence of correlation between *P. gingivalis* and AS

### 2.1 Clinical studies

The American Heart Association (AHA) supports an independent association between periodontal disease (PD) and atherosclerotic vascular disease, but not a causal relationship between the two (19). Subsequent studies in the last decade also lack evidence to date for a causal relationship between PD and AS. Current clinical studies have mainly elaborated on the correlation between PD and AS by detecting the colonization of periodontal pathogens in AS plaques and serum levels of AS-associated inflammatory markers.

Clinical studies have shown that abundant *P. gingivalis* colonization can be detected in AS plaques of different artery types. A Meat analysis based on 1791 patients showed that *P. gingivalis* specifically localized to coronary AS plaques but not to other organs (20). A study of 58 patients with moderate or severe periodontitis with AS using a 16sRNA assay found *P. gingivalis* detection rates of 26.7% in carotid arteries and 39.3% in coronary arteries (21). Also using the detection method of 16sRNA assay, some studies have pointed out that the detection rate of periodontal pathogens including *P. gingivalis* in carotid AS lesions is only 21% (22). Another study using metagenomics techniques noted that *P. gingivalis* colonization was detected in the coronary or femoral arteries of 42 patients with AS who participated in the study and that *P. gingivalis* accounted for nearly 80% of all colonized bacterial species, but the study was notably deficient in that it did not assess the periodontal status of the participants (23). Some scholars believe that the difference in the detection rate of *P. gingivalis* in AS plaques may be due to demographic, geographical, and ethnic differences, as well as different sampling methods and laboratory testing methods of clinical samples, but there are no related statistical and methodological differences (24). The colonization and invasion of *P. gingivalis* into the arterial wall allows *P. gingivalis* to acquire a “privileged niche”. This “privileged niche” not only helps *P. gingivalis* to obtain proteins and iron substrates from the host but also separates *P. gingivalis* from humoral and cellular immunity, thus inducing endothelial dysfunction and promoting AS progression (25) (**Table 1**).

**Table 1:** Clinical Research Evidence of *P. gingivalis* and AS.

Research Type	Country	Research Object	Sample Type	Result	References
Meta analysis	/	1791 AS patients	Coronary artery, carotid artery	*P. gingivalis* is one of the bacteria peculiar to AS plaque	(20)
Clinical research	America	42 AS patients	Coronary artery, femoral artery	*P. gingivalis* accounts for nearly 80% of all bacterial species colonized in AS plaque	(23)
Clinical research	Serbia	58 AS patients with periodontitis	Coronary artery, carotid artery	*P. gingivalis* was detected in 26.7% of carotid arteries and 39.3% of coronary arteries	(21)
Clinical research	France	45 AS patients with periodontitis	Carotid artery	The detection rate of periodontal pathogens including *P. gingivalis* in AS lesions was only 21%	(22)
Clinical research	Sweden	42 healthy people, 89 periodontitis patients	Serum	The IgG antibody titer of anti-*P. gingivalis* in the serum of patients with periodontitis is at a long-term stable high level	(26)
Clinical research	Columbia	22 patients with gingivitis, 22 patients with periodontitis	Serum	Serum E-selectin, MPO, and ICAM-1 levels in patients with periodontitis increased	(27)
Clinical research	Britain	120 patients with severe periodontitis	Serum	Periodontal treatment reduces the level of inflammatory factors in the blood and improves the endothelial function of arteries	(28)
Clinical research	Brazil	69 patients with coronary heart disease severe and periodontitis	Serum	Periodontal treatment maintained a relatively normal blood concentration of markers of vascular inflammation but did not improve arterial vascular conditions	(29)
Clinical research	South Korea	247696 healthy adults aged 40 and over	/	Daily brushing and regular dental maintenance reduce AS risk	(30)
Clinical research	America	15792 participants aged 45-64	/	Regular dental care and care significantly reduce the risk of AS	(31)
Clinical research	America	8999 participants aged 20-85	/	The incidence rate of cardiovascular diseases in patients with poor periodontal treatment prognosis is 1.28 times higher than that in patients with a good prognosis	(32)

In patients with periodontitis, the presence of *P. gingivalis* colonization in the arterial vessel wall, as well as significantly higher serum anti-*P. gingivalis* antibody titers (26, 33) and inflammatory factor levels (27, 34) may lead to a concomitant increase in AS risk (35, 36). In contrast, routine oral maintenance and periodontal treatment significantly reduced the levels of AS-related inflammatory factors in the serum of the patient and significantly improved the endothelial function of the patient’s arterial vessels, leading to a consequent reduction in the risk of AS.

Several recent large population studies have evaluated the effect of routine oral maintenance and periodontal treatment on the progression of AS, and have reached similar conclusions. A Korean follow-up study based on 247,696 healthy adults aged 40 years and older showed that brushing more than once a day and regular professional dental cleanings reduced the risk of cardiovascular events, including AS, by 9% and 14%, respectively (30). A U.S. study of atherosclerosis risk in communities (ARIC) based on 15,792 participants aged 45-64 years over 15 years showed that regular dental care and attention significantly reduced the risk of AS (31). Another U.S. study based on 8,999 participants aged 20-85 years with 16.8 years of follow-up noted that the incidence of cardiovascular disease, including AS, was 1.28 times higher in those with a poor prognosis for periodontal treatment than in those with a good prognosis for periodontal treatment (32). A study of 120 patients with severe periodontitis showed lower levels of inflammatory factors in the blood after 24 hours of periodontal treatment compared to usual oral care, and a significant improvement in endothelial function of the arteries at six months (28). Plasma levels of AS-related risk molecules, including inflammatory factors (CRP, IL-6, TNF-α), thrombotic molecules (fibrinogen), and metabolic markers (triglycerides, TC, HDL-C, HbA1c, A-FABP), were significantly reduced within six months after periodontal treatment means to eliminate periodontal inflammation (37–40). However, it has also been shown that periodontal treatment, while maintaining relatively normal blood concentrations of vascular inflammatory markers, did not improve arterial vascular status in a short-term follow-up within three months after periodontal treatment and did not indicate an AS risk-reducing effect (29) (**Table 1**).

Based on the above clinical basis, we can speculate that *P. gingivalis* may promote AS by colonizing the arterial wall and causing abnormal serum inflammatory factor levels; however, there is still insufficient evidence from clinical studies on *P. gingivalis* and AS. Comparative studies on the colonization of *P. gingivalis* in the arterial canal wall before and after periodontal treatment are lacking. Current epidemiological data focus on medium-sized arteries such as coronary, carotid, and femoral arteries, however, the lack of studies on large arteries such as the aorta may be significantly associated with the availability of experimental samples. In addition, PD and AS each have multiple complex and critical risk factors that are pervasive and powerful contributors and difficult to fully exclude, including age, smoking, and diabetes mellitus (41). Therefore, future studies should increase the consideration of multiple confounding factors, including the criteria for admission to PD, standardized treatment regimens for PD, recurrence of PD and evolution of AS, and other important risk factors.

### 2.2 Animal studies

Animal studies have shown that ApoE^-/-^ mice with oral *P. gingivalis* infection have elevated serum levels of cellular inflammatory factors (42) and significantly increased vascular reactivity (43), and *P. gingivalis* was detected to colonize the aorta, damaging the arterial endothelium (44) and increasing AS plaque (45). In addition, numerous studies have demonstrated that serum inflammatory mediators and lipoprotein levels were significantly abnormal in oral *P. gingivalis*-infected ApoE^-/-^ mice, such as NLRP3, IL-6, IL-1β, TNF-α, intercellular adhesion molecule-1 (ICAM-1), vascular adhesion molecule 1 (VCAM-1), monocyte chemotactic protein(MCP), P-selectin, E-selectin, low-density lipoprotein (LDL), HDL, etc (46, 47) (**Table 2**).

**Table 2:** Animal Research Evidence of *P. gingivalis* and AS.

Time	Animal Type	Modeling method	Sample Type	Result	References
2011	C57BL/6 ApoE^-/-^ mice	Oral infection with *P. gingivalis*	Serum, spleen	Promote the release of systemic pro-inflammatory cytokines and accelerate the progress of AS	(42)
2011	C57BL/6 wild-type, ApoE^-/-^ mice	Oral infection with *P. gingivalis*	Serum, aorta	Enhance arterial reactivity mediated by α-adrenoceptor	(43)
2014	ApoE^null^ B6.129P2-Apoe^tm1Unc^/J mice	Oral infection with *P. gingivalis*	Serum, aorta	Promote the colonization of viable aortic bacteria and increase inflammation	(45)
2015	ApoE^-/-^ B6.129P2-Apoe^tm1Unc^/J mice	Oral infection with complex (*P.g*,*T.d*,*T.f*,*F.n)*	Serum, aorta	Disturb the serum lipid profile and promote the progress of AS	(46)
2015	C.KOR Apoe^shl^ mice	Oral infection with *P. gingivalis*	Serum, aorta	Increase the area of aortic plaque significantly, NLRP3 inflammasome played an important role	(47)
2021	C57BL/6 ApoE^-/-^ mice	Oral infection with *P. gingivalis*	Serum, aorta, liver	Promote vascular endothelial dysfunction	(44)
2010	ICR mice	Intravenous injection with *P. gingivalis*	Femoral artery	Promote the hyperplasia of arterial intima, and is related to the over-expression of S100A9 and SMemb on the surface of smooth muscle cells	(48)
2016	C57BL/6J wild-type mice	Subcutaneous inoculation with *P. gingivalis*、ligation of coronary artery	Heart	Increase the risk of heart rupture significantly	(49)
2016	B57BL/6 wild-type, B57BL/6-Tlr4^lps-del^ mice	Intravenous injection with *P. gingivalis* GroEL	Serum, aorta	Increase expression of VCAM-1, ICAM-1, TLR4, and LOX-1 in the aorta	(50)
2017	C57BL/6 ApoE^-/-^ mice	Intravenous injection with *P. gingivalis*	Serum, aorta, heart, liver	Abnormal lipid profiles were found in serum, heart, aorta, and liver	(51)
2020	C57BL/6 ApoE^-/-^ mice	Intravenous injection with *P. gingivalis*	Serum, aorta	Increase expression of oxidative stress markers and inflammatory factors in serum and aorta	(52)
2014	C.KOR-Apoe^shl^ mice	Sublingual immune with *P. gingivalis* rGroEL	Serum, aortic, spleen	Reduce AS lesions in the aortic sinus significantly, and decrease serum levels of CRP, MCP-1, and ox-LDL.	(53)
2018	LDLR^-/-^ mice	subcutaneous injection with *P. gingivalis* Rgp44	Serum, aorta	Generate protective IgM for ox-LDL to reduce AS risk	(54)
2020	ApoE^-/-^ mice	Nasal Immune with *P. gingivalis* GroEL Derived Pep14	Serum, aorta, liver	Reduce AS risk by promoting IFN- γ secretion and/or inhibiting Th17-mediated immune response	(55)
2021	ApoE^-/-^ mice	Nasal immune with inactive *P. gingivalis*	Aorta	Reduce plaque area of AS significantly, and the effect was similar to that of statins	(56)

In addition to oral infection with *P. gingivalis*, some studies have also infected mice by subcutaneous inoculation or intravenous *P. gingivalis* injection. Animal studies have shown that subcutaneous inoculation with *P. gingivalis* accelerates AS and leads to a significant increase in mortality from cardiac rupture (49). Intravenous *P. gingivalis* injection revealed significant intimal hyperplasia and VSMCs proliferation in the aorta of mice (48). After intravenous *P. gingivalis* injection, ApoE^-/-^ mice showed increased expression of oxidative stress markers and inflammatory factors in serum and aorta (52), and abnormal lipid profiles were found in serum, heart, aorta, and liver (51). Intravenous injection of recombinant *P. gingivalis* heat shock protein GroEL into C57BL/6 mice on a high-fat diet resulted in stronger expression of VCAM-1, ICAM-1, TLR4, and lectin-like ox-LDL receptor (LOX-1) (50) (**Table 2**).

Some recent studies have also conducted experiments on the relationship between *P. gingivalis*-related vaccines and AS. Heated ultrasound *P. gingivalis*-prepared vaccine significantly reduced AS plaque area in ApoE^-/-^ mice on the background of high-fat diet and oral *P. gingivalis* infection employing nasal immunization, with effects comparable to those of statins (56). Similarly, sublingual immunization with *P. gingivalis* GroEL (53) or nasal immunization with its derivative peptide 14 (Pep14) (55) achieved inhibition of AS plaque formation. In addition, subcutaneous immunization of LDLR^-/-^ mice with the A hemagglutinin domain (Rgp44) of *P. gingivalis* promotes the production of protective IgM against ox-LDL and reduces the risk of AS (54). There are few studies on the relationship between *P. gingivalis*-related vaccines and AS, and the mechanism of action is not fully understood and needs to be further investigated (**Table 2**).

Oral infection with *P. gingivalis* can lead to dysregulated intestinal flora (57), and dysregulated intestinal flora can also promote AS progression through metabolism-dependent pathways (58). Among them, Trimethylamine-N-oxide (TAMO) is one of the most important metabolites associated with dysregulated intestinal flora (59, 60). TMAO promotes foam cell production by upregulating macrophage CD36 and SR-A1 expression which impairs macrophage cholesterol reversal transport function (61). TMAO promotes the release of pro-inflammatory mediators through the activation of mitogen-activated protein kinase, extracellular signal-associated kinase, and NF-κB cascade pathways, which in turn induces an inflammatory response in ECs and VSMCs (62–64). TMAO exacerbates AS progression by promoting the release of intracellular Ca^2+^, leading to platelet aggregation and thrombosis (65).

However, there are still limitations in the establishment of current animal models. Intravenous injection of planktonic state *P. gingivalis* in an infectious manner not only hardly mimics the characteristics of inflammation triggered by normal plaque biofilm but also may lead to a sharp increase in the level of *P. gingivalis* in the circulatory system and trigger a strong stress response. Thus, the type and degree of the inflammatory response induced by intravenous *P. gingivalis* may not match the chronic state of infection in a real situation. Certainly, the intravenous approach may apply to the study model of acute systemic bacterial infections.

It is noteworthy that both clinical and animal studies have used 16srRNA, metagenomics sequencing to detect *P. gingivalis* g gene fragments in the arterial wall to determine colonization. But these techniques are impossible to assess the activity status and reproduction of *P. gingivalis* after colonization or to determine whether the gene fragments are from intact bacteria or *P. gingivalis* OMVs. These are limitations of the current assays. The activity of *P. gingivalis* colonized in the arterial wall or the different virulence factors released by dead bacteria may directly influence the degree of local inflammatory response in the vessel wall. *In vitro* studies have pointed out that live *P. gingivalis* induces monocyte adhesion to the endothelium and promotes the vascular inflammatory response by promoting the expression of adhesion molecules and pro-inflammatory factors in ECs, whereas heat-killed *P. gingivalis* does not trigger these effects (66, 67). Therefore, the conclusion that *P. gingivalis* colonizes the arterial vessel wall cannot yet be directly correlated with vascular inflammation using these techniques. Further culture and characterization of strains of AS tissue are needed to explore *P. gingivalis* activity after colonization. This is a central part of the follow-up study.

## 3 Pathogenicity of *P. gingivalis* and pathogenesis of AS

### 3.1 Pathogenicity of *P. gingivalis*

*P. gingivalis* is the “dominant flora” in periodontitis, which can reshape the symbiotic colonization of periodontal tissues and induce dysbiosis of periodontal microbial homeostasis (2). During disease development, *P. gingivalis* interacts with the host immune system through its unique virulence factors, resulting in a unique and complex pathogenic mechanism, such as induction of inflammatory response, activation of the complement system, promotion of apoptosis, and other biological processes (1). In addition, the virulence factors of *P. gingivalis* can interact with various host receptors to reshape the survival environment or escape host immune killing, allowing them to persist in host tissues (3).

#### 3.1.1 *P. gingivalis* gingipains

*P. gingivalis* gingipains have potent proteolytic activity and play a key role in disrupting the host immune response. *P. gingivalis* gingipains include arginine-specific gingipains (RgpA and RgpB), and lysine-specific gingipains (Kgp). *P.* gingivalis gingipains cause host immune dysregulation and inflammatory responses to occur by activating matrix metalloproteinases, inactivating immunosuppressants, degrading immunomodulatory factors, and cleaving immune cell receptors (6). *P. gingivalis* gingipains degrade the junctional adhesion molecule (JAM1) of gingival epithelial cells, disrupting epithelial barrier function and increasing the ability of bacteria and their products such as LPS and peptidoglycan (PGN) to locally invade and penetrate the peripheral blood (68). *P. gingivalis* gingipains selectively degrade the macrophage surface innate immune receptor CD14, resulting in the hyporesponsiveness to bacterial challenge (69). *P. gingivalis* gingipains can degrade neutrophil-derived α-defensins and β-defensins, disrupting the host’s innate immune function and facilitating bacterial escape (70). In addition, *P. gingivalis* gingipains have a significant disruptive effect on the complement system. On the one hand, *P. gingivalis* gingipains inhibited the bactericidal effect of the complement system by degrading C3, C4, and C5 to inhibit complement activation and the formation of membrane attack complexes (71). On the other hand, *P. gingivalis* gingipains release the allergenic toxin C5a by cleaving complement C5, causing stronger inflammation (72). C5a induced by *P. gingivalis* gingipains can also subtly evade immune clearance *via* the C5aR-TLR2 crosstalk pathway (73).

#### 3.1.2 *P. gingivalis* LPS

*P. gingivalis* LPS consists of lipid A, core oligosaccharide, and O-specific polysaccharide (6). *P. gingivalis* LPS can trigger innate immune responses by activating TLRs (74). The virulence properties of *P. gingivalis* LPS are determined by lipid A properties, thus different properties of lipid A can lead to different innate immune responses and the production of inflammatory factors. *P. gingivalis* LPS_1690_ with penta-acylated lipid A mainly activates the NF-κB signaling pathway, while *P. gingivalis* LPS_1435/1449_ with tetra-acylated lipid A mainly induces p38/MAPK and ERK1/2 signaling pathways (74). *P. gingivalis* LPS induces M1-type polarization in macrophages and promotes the expression of several pro-inflammatory factors, such as TNF-α, IL-1β, and IL-6 (75). *P. gingivalis* LPS promotes the progression of periodontal inflammation by inducing pyroptosis in gingival fibroblasts (76). *P. gingivalis* LPS triggers mitochondria-mediated apoptosis by regulating XBP1 expression (77). *P. gingivalis* LPS promotes platelet proliferation and thrombosis by activating platelet Cdc42 (78).

#### 3.1.3 *P. gingivalis* fimbriae

The fimbriae of *P. gingivalis* are divided into long fimbriae (FimA) and short fimbriae (Mfa1), both of which have enhanced inflammatory responses and evasion of host immune clearance, although each has its mechanism of action with the host. FimA acts through the characteristic *P. gingivalis* peptidilarginine deiminase (PPAD)-dependent activation of TLR2, induction of NF-ĸB and MAPK signaling pathways, and promotion of pro-inflammatory factor production (79). FimA interacts with complement receptor 3 (CR3) in macrophages, leading to ERK1/2 phosphorylation and inhibition of IL-12 production to promote the survival of *P. gingivalis* (80). FimA induces cAMP-dependent protein kinase A (PKA) activation *via* instigating macrophage CXCR4/TLR2 co-association, which in turn inhibits TLR2-mediated antimicrobial responses (81). The binding of FimA to CXCR4 induces CR3 activation *via* phosphatidylinositol-3 kinase (PI3K) and inhibits the antibacterial response in macrophages (82). Similar to FimA, Mfa1 induces the production of pro-inflammatory factors through the activation of TLRs (83, 84). Mfa1 inhibits the autophagy of DCs through the DC-SIGN-TLR2 crosstalk pathway, evading intracellular killing and leading to long-term survival within DCs (85).

### 3.2 Pathogenesis of AS

AS is a chronic cardiovascular disease that threatens human health and is characterized by lipid deposition in parts of the artery, accompanied by VSMCs and fibrous matrix proliferation, which gradually forms an AS plaque. AS is often considered a chronic inflammatory disease because inflammation plays an important role in all stages of AS development (86). The AS-associated inflammation is mainly mediated by pro-inflammatory factors, inflammatory signaling pathways, bioactive lipids, and adhesion molecules (87).

The main triggers of AS are endothelial damage, abnormal lipid metabolism, and hemodynamic impairment. In the early stages of AS, these pathological factors activate ECs (88). When ECs are activated, they express a variety of pro-inflammatory factors and adhesion molecules, including MCP-1, IL-8, ICAM-1, VCAM-1, E-selectin, and P-selectin, which attract lymphocytes and monocytes that bind to ECs and infiltrate the arterial wall, promoting the progression of the inflammatory response (89). Among them, pro-inflammatory monocytes expressing high levels of Ly6C or Gr-1 preferentially accumulate at damaged endothelial sites (90). Immune cells residing in the vessel wall participate in the inflammatory response process in the vessel wall together with the attracted immune cells. Large amounts of LDL are modified to ox-LDL and accumulate in the vessel wall, while macrophages in the vessel wall take up ox-LDL and convert it to foam cells, leading to the formation of AS plaques (91). Other types of immune cells, such as dendritic cells (DCs), T cells, B cells, and neutrophils are also involved in the progression of the inflammatory response within the plaque (92). In the advanced stages of AS, large numbers of macrophages and pro-inflammatory factors infiltrate the vessel wall, secrete matrix metalloproteinases (MMPs), and degrade collagen fibers in the extracellular matrix, leading to plaque rupture, hemorrhage, and thrombosis (89).

### 3.3 Pathogen-associated molecular patterns and damage-associated molecular patterns play a “bridging” role between *P. gingivalis* and AS

PAMPs are conserved pathogenic molecular structures shared by pathogenic microorganisms, while DAMPs are substances released into the intercellular space or blood circulation upon stimulation of tissues or cells (93). These substances bridge the gap between periodontitis and AS, allowing us to further understand the relationship between oral and systemic diseases (94).

*P. gingivalis* contains multiple PAMPs, including LPS and PGN, which initiate the inflammatory response of innate immunity by relying on the recognition of host cell pattern-recognition receptors (PRRs), such as NLRs and TLRs. *P. gingivalis* LPS induces the release of pro-inflammatory factors by activating NF-κB and MAPK signaling pathways in macrophages (95). *P. gingivalis* LPS promotes high expression of angiotensin II (Ang II) and IL-6 in ECs and accelerates ECs dysfunction (67). *P. gingivalis* LPS promotes monocyte chemotaxis and adhesion by increasing the expression of chemotactic and adhesion molecules in ECs through Akt and NF-κB signaling pathways (96). PGN promotes ICAM-1 production by monocytes through the activation of TLR2 and NF-κB pathways and induces monocyte migration and adhesion to the vascular endothelium (97). PGN promotes the upregulation of VCAM-1 through the NOD1-RIP2-NF-κB axis, inducing myeloid cells to recruit to the endothelium and leading to endothelial dysfunction (98). PGN can also mediate the over-expression of adhesion molecules in ECs through innate peptidoglycan recognition protein 1 (99). PGN induces the production of pro-inflammatory cytokines through TLR2 and CD14 and increases the susceptibility of AS plaques (100).

Meanwhile, periodontal pathogens further promote the progression of AS by activating inflammatory caspases that induce cell death and release various DAMPs, such as HSP60, cardiolipin, alarmins (S100 protein), and high mobility group box 1 (HMGB1) (101). HSP60 promotes immune cell migration and adhesion to the endothelium by stimulating the expression of E-selectin, VCAM-1, and ICAM-1 in ECs, and also induces endothelial inflammatory responses by activating TLRs (mainly TLR4) in innate immune cells (macrophages, DCs) (102). HSP60 induces DCs maturation and activates Th1 and Th17 cells in an MHC-II-dependent manner, promoting the release of pro-inflammatory mediators (103). HSP60 induces the activation of specific CD4^+^CD25^+^CD45RO^+^ T cells, which bind to ECs expressing HSP60 and adhesion molecules (VCAM-1 and E-selectin), forming susceptible sites of AS lesions (104). HSP60 induces the proliferation of VSMCs *via* TLR2 and TLR4 (105). The released cardiolipin may be oxidized by *P. gingivalis* to become oxidized cardiolipin (106, 107). Oxidized cardiolipin increases the expression levels of ICAM-1 and VCAM-1 in ECs and induces migration and adhesion of immune cells. At the same time, oxidized cardiolipin could also activate 5-lipoxygenase and induce leukotriene B4 production by increasing intracellular calcium concentration in macrophages and neutrophils, promoting inflammatory responses and exacerbating AS progression (108). *P. gingivalis* could promote VSMCs from contractile phenotype to synthetic phenotype by upregulating the expression of S100A9 in VSMCs (109). The circular RNA PPP1CC of *P. gingivalis* could promote VSMCs pyroptosis through the HMGB1/TLR9/AIM2 axis, which in turn increases AS plaque vulnerability (110).

## 4 Immune mechanism of *P. gingivalis* to promote AS progression

Immunity is an important line of defense of the organism against pathogenic invasion. *P. gingivalis* expresses a variety of virulence factors that stimulate host immune responses and play an important role in promoting AS progression. On the one hand, *P. gingivalis* evades host innate and adaptive immunity, internalizes in host tissues, and, in various forms, circulates with blood and lymph and colonizes arterial vessel walls, causing local inflammation and lesions; on the other hand, *P. gingivalis* persistently stimulates host immune responses in the process of host immune evasion and systemic dissemination, inducing systemic inflammatory mediators and disruption of lipid levels, and promoting AS progression. Based on the possible key role of *P. gingivalis*-associated PAMPs and DAMPs in promoting AS progression, the following literature will be reviewed from three aspects involving immune response: immune escape, blood circulation, and lymphatic circulation.

### 4.1 Immune escape

Immune escape is an important pathway for *P. gingivalis* to invade and survive in the host for a long time. A recent paper has reviewed *P. gingivalis* evasion of host immune killing through various pathways but did not elucidate the possible potential association and mechanism of *P. gingivalis* with AS during immune evasion (111). In this section, we will review the potential associations and mechanisms of *P. gingivalis* with AS during immune evasion in terms of dysregulation of the complement system and disruption of immune cell function.

#### 4.1.1 *P. gingivalis* interferes with the function of the complement system

The complement system is a major part of the innate immune system and is activated by the hydrolytic cascade reaction of serine proteases. In inflamed vessels, the complement system can be activated by conjugates of CRP and modified LDL (112), inducing apoptosis of ECs, promoting the proliferation of VSMCs, inducing the release of procoagulant and adhesive factors, recruiting immune cells, and accelerating AS progression (113).

Studies suggest that the virulence factors of *P. gingivalis* may promote AS progression by interfering with the function of the complement system and, in turn, by promoting AS progression. *P. gingivalis* gingipains cleave complement C5 into biologically active C5a and C5b fragments (114, 115) and induce M1-type polarization of macrophages *via* the C5a pathway to promote inflammatory factor secretion (116). C5a is present in AS plaques and acts as a pro-AS molecule (117–119). In the early plaque formation stage, C5a activates mast cells in the arterial wall, promotes secretion of fibrinogen activator inhibitor (PAI-1), inhibits fibrinolysis and extracellular protein hydrolysis, and accelerates thrombus and AS plaque formation (120, 121). In the advanced stage of AS, C5a induces apoptosis of ECs and VSMCs, and expression of MMP1 and MMP9 in plaques, leading to VSMCs-dependent collagen loss, fibrous cap thinning, and plaque rupture (118, 122, 123). In addition, C5a accumulation enhanced NLRP3 inflammatory vesicle activation in AS plaques and decreased plaque stability (124). In contrast, a significant reduction in AS plaque area was observed after treatment of ApoE^-/-^ mice with C5aR antagonists (125, 126). Notably, *P. gingivalis* cleavage of C5 produced large amounts of C5a, which activated C5aR, triggered cross-talk signal between TLR2 and C5aR (127), suppressed macrophage immune function, increased *P. gingivalis* survival, and led to ubiquitinated degradation of myeloid differentiation factor (Myd88) (127–129), which is also a positive regulator of foam cell formation in AS (130). Therefore, it is speculated that the crosstalk between TLR2-C5aR may be closely related to the promotion of foam cell formation by *P. gingivalis*. However, it has not been reported yet and needs further in-depth study.

**Figure 1:**
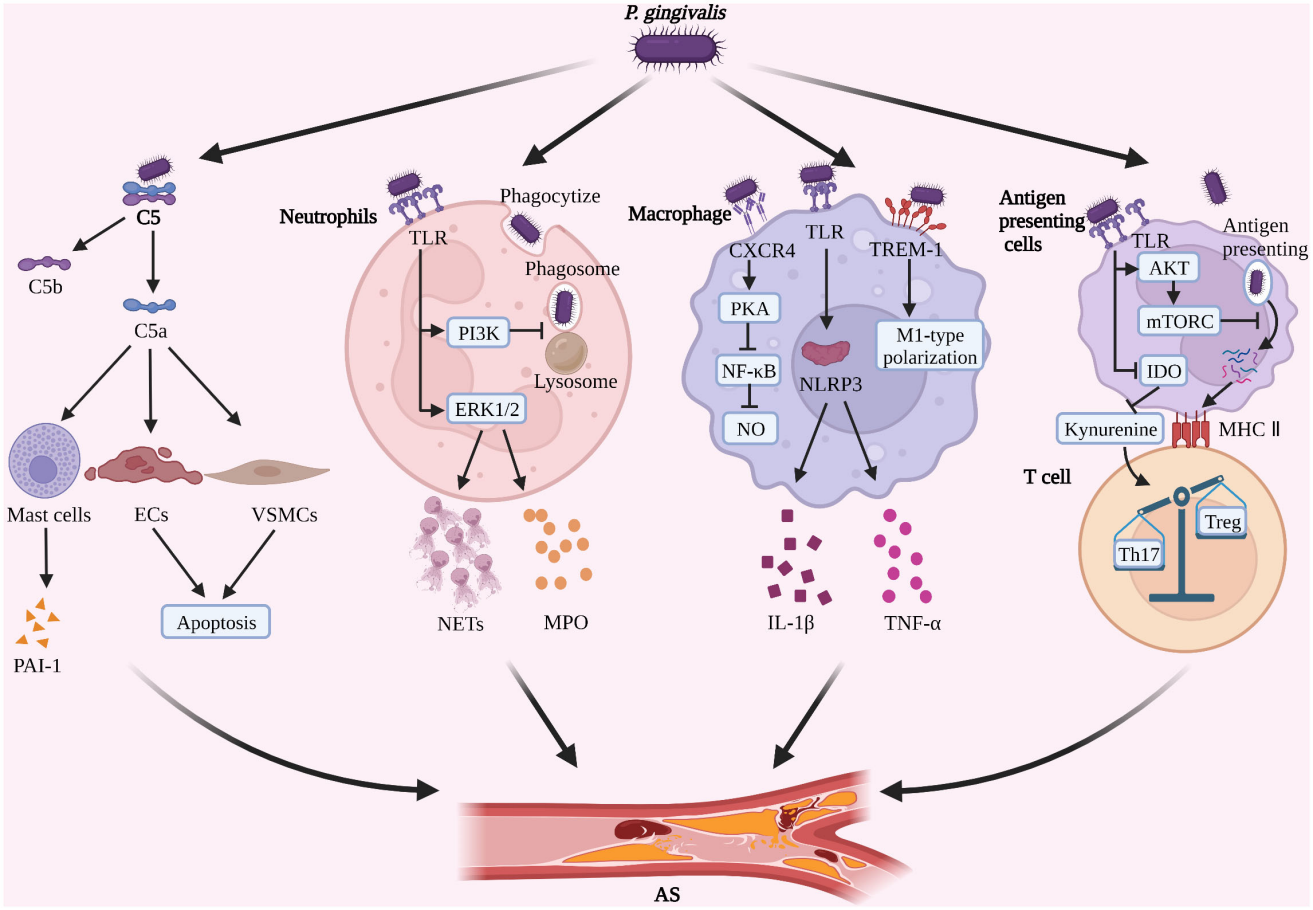
*P. gingivalis* induces immune escape and promotes AS progression (1). *P. gingivalis* degrades complement C5, promotes PAI-1 secretion by mast cells, and induces apoptosis of ECs and VSMCs (2). *P. gingivalis* inhibits phagosome-lysosome fusion in neutrophils, induces the formation of NETs, and increases secretion of MPO (3). *P. gingivalis* inhibits NO formation in macrophages and promotes M1-type polarization and activation of NLRP3 (4). *P. gingivalis* inhibits the antigen presentation process and induces Th17/Treg imbalance. (Created with BioRender.com).

The above studies suggest that *P. gingivalis* gingipains may promote AS plaque formation and rupture by interfering with complement C5a function (**Figure 1**). However, the current studies on the promotion of AS development by *P. gingivalis* through interference with the complement system are very limited, mainly focusing on the effects of *P. gingivalis* gingipains with C5a. However, whether similar effects and mechanisms exist for other virulence factors of *P. gingivalis*, whether other components of the complement system are involved in AS progression, and whether *P. gingivalis*-mediated TLR2-C5aR crosstalk is associated with foam cell formation is not fully understood and need to be further investigated in depth.

#### 4.1.2 *P. gingivalis* inhibits the antimicrobial function of immune cells

*P. gingivalis* inhibits the phagocytosis, surveillance, and clearance functions of immune cells through various mechanisms, evades host immune killing, survives in the host for a long time, repeatedly stimulates the body’s immune system, leads to a persistent low-level inflammatory state in the host, and promotes the progression of AS.

##### 4.1.2.1 Neutrophils

Neutrophils are a class of innate immune cells that are the first to reach the site of *P. gingivalis* infection and can constitute an important barrier against *P. gingivalis* by producing proteases, antimicrobial peptides, and extracellular traps (NETs) (131, 132), as well as being important regulators of vascular inflammation (133).

It was found that *P. gingivalis* can evade immune killing by neutrophils. *P. gingivalis* activates the non-MyD88-dependent TLR2-PI3K signaling pathway in neutrophils, which both reduces the phagocytosis of *P. gingivalis* by neutrophils and blocks intracellular phagosome-lysosome fusion, thereby increasing the intracellular survival of *P. gingivalis* (129). It was also noted that *P. gingivalis* stimulated neutrophils to form NETs in a gingipains-dependent manner, but the antimicrobial components (histone protease, LL-37, etc.) in the formed NETs were hydrolyzed by gingipains, resulting in the lack of antimicrobial activity of NETs and the inability to achieve *P. gingivalis* clearance (132). In addition, the OMVs secreted by *P. gingivalis* can coat the neutrophils without being internalized, while the gingipains carried can degrade LL-37 and myeloperoxidase (MPO), which have secreted antimicrobial particle activity, and thus achieve the effect of avoiding neutrophil killing from a distance (134). The above pathways provide an opportunity for *P. gingivalis* to evade neutrophil immune killing, however, the efficacy of the various pathways has not been conclusively established.

However, it should not be overlooked that *P. gingivalis* still has a continuous stimulatory effect on neutrophils after evading immune killing, causing neutrophils to develop immune tolerance and inhibiting phagocytosis of *P. gingivalis*, while possibly promoting the progression of AS. *P. gingivalis* LPS-tolerant neutrophils have reduced phagocytosis of *P. gingivalis*, but significantly increased NETs formation and increased extracellular MPO levels, which may be related to neutrophils’ immune reconstitution (135). However, as already mentioned, *P. gingivalis* gingipains can degrade MPO (134). The “antagonism” between the pathogenicity of evaded phagocytosed *P. gingivalis* and the antimicrobial capacity of tolerant neutrophils determines the final fate of *P. gingivalis*. Furthermore, it has been shown that excess NETs promote plaque instability by directly inducing the death of ECs (136, 137), and that plasma MPO levels are positively correlated with the risk of AS (138). It is suggested that *P. gingivalis* may accelerate the progression of AS by promoting the production of NETs and MPO by tolerogenic neutrophils (**Figure 1**). *P. gingivalis* gingipain R and some host-derived proteases inhibit the nonphlogistical clearance of apoptotic cells by macrophages through hydrolytic modification of apoptotic neutrophil surface protein ligands, leading to local accumulation of apoptotic neutrophils and secondary necrosis (139). The process of nonphlogistical clearance of apoptotic cells by macrophages is called “efferocytosis” and defective efferocytosis exacerbates AS progression (140).

##### 4.1.2.2 Macrophages

Macrophages are a class of intrinsic immune cells with strong phagocytic capacity and antigen-presenting function, which can exert antimicrobial effects by phagocytosing *P. gingivalis* and *P. gingivalis*-infected cells, secreting pro-inflammatory cytokines (141), and are also major players in the formation of foam cells and mediating AS plaque stability (142).

It was found that *P. gingivalis* can undergo repeated cycling behavior in and out of cells in macrophages and successfully avoid macrophage killing (143), which may be related to the following mechanisms. First, the high heme concentration in the inflammatory environment can convert *P. gingivalis* surface lipid A to a tetraacylated form without TLR4 agonistic activity, limiting macrophage activation (144–146). Second, various specific virulence factors of *P. gingivalis* play an important role in interfering with macrophage immune responses. *P. gingivalis* gingipains degrade caspase-1, IL-1β, and CD14, inhibit the activation of a TLR2/4 signaling pathway in macrophages (69, 147), and suppress the bactericidal effect of inflammasomes (148). *P. gingivalis* FimA (long or major fimbriae) bind to CXCR4, both through PI3K signaling activates CR3 on macrophages contributing to *P. gingivalis* immune evasion (82, 149), and also induces cAMP-dependent protein kinase A (PKA) signaling, inhibits TLR2/1-mediated NF-κB activation and NO production, suppresses macrophage activation, and improves *P. gingivalis* survival and virulence (81). *P. gingivalis* sialidase inhibits macrophage IL-12 expression (150), thereby suppressing NK cell activation and the Th1 to Th2 phenotype switch, which in turn reduces the clearance of *P. gingivalis* (151). In addition, besides inhibiting the endocytic digestion of macrophages, *P. gingivalis* can also inhibit the autophagy of macrophages to a certain extent, leading to the immune escape of *P. gingivalis*. It has been shown that *P. gingivalis* can induce autophagy in macrophages and promote the clearance of *P. gingivalis* by macrophages (152). However, it has also been shown that different *P. gingivalis* LPS variants (LPS_1690_, LPS_1435/1449_) exert different promoting or inhibiting effects on autophagy. Among them, the dominance of different *P. gingivalis* LPS variants depend on factors such as temperature, growth cycle, and heme chloride level (5, 153). LPS_1690_ induces macrophages to produce giant LC3-positive vesicles and melanoregulin puncta (cargo sorting protein), promoting lysosome maturation and autophagic response, while the presence of LPS_1435/1449_ significantly inhibited the above effects (154). Therefore, it is speculated that the different occupancy of LPS_1690_ and LPS_1435/1449_ in *P. gingivalis* may regulate the autophagy of macrophages and thus affect the survival of *P. gingivalis*. However, this has not been reported and needs to be further investigated in depth.

*P. gingivalis*, which survives in macrophages, induces inflammatory vesicle activation and M1-type polarization in macrophages (155). Animal studies have shown that oral inoculation of *P. gingivalis* can activate NLRP3 inflammasomes in macrophages in AS plaques in a gingipains-independent manner, promote the release of IL-1β and TNF-α, and accelerate AS progression (47, 148). *In vitro* studies have revealed that *P. gingivalis* LPS may induce M1-type polarization of macrophages *via* pattern recognition receptor triggering receptors expressed on myeloid cells-1(TREM-1) and its downstream signaling pathways to promote the secretion of multiple inflammatory factors and accelerate AS progression (75, 156, 157) (**Figure 1**). However, recent studies have also indicated that *P. gingivalis* LPS-induced tolerant macrophages represent an intermediate state between M1/M2 polarization and function as M2-like cells to limit the inflammatory response (158), suggesting that *P. gingivalis* LPS tolerant macrophages may be closely related to tissue repair. This may be related to the different macrophage sources, stimulation factors, and temporal phases, and needs to be analyzed in further studies. In addition, the foam-like, anti-inflammatory TREM2^hi^ macrophage subpopulation identified by recent single-cell sequencing studies may be associated with plaque calcification (9). However, no studies related to *P. gingivalis* and TREM2^hi^ macrophage subsets have been reported. As a key cell type in host immune response and AS plaque formation, macrophages may have important intersections with multiple immune and metabolic mechanisms, but there is no uniform conclusion yet, and further in-depth studies are needed.

##### 4.1.2.3 T cells

T cells are the main performers of adaptive immunity in the body and are also important regulators of AS plaque formation, development, and late stability (159). Notably, the hypoxic and ischemic environment of AS plaques may also have an impact on T cell metabolic status and function, which in turn affects AS progression (160, 161).

It is suggested that *P. gingivalis* may evade T cell immune killing by inhibiting antigen presentation function and thus T cell activation, proliferation, and antimicrobial function. In terms of inhibiting antigen presentation, *P. gingivalis* promotes IL-10 secretion by macrophages, downregulates the expression of MHC-II molecules (162), and promotes PD-L1 and PD-1 binding on the surface of macrophages and CD4^+^ T cells, inhibiting antigen presentation and T cell activation (162–164). In addition, *P. gingivalis* interferes with DCs autophagy and apoptotic processes by activating the Akt/mTOR axis, thereby promoting survival in DCs and inhibiting *P. gingivalis* processing and presentation by DCs (165, 166). In addition to inhibiting antigen presentation function, *P. gingivalis* can also directly inhibit T cell activation and proliferation. *P. gingivalis* and its Rgp-gingipains inhibit T cell proliferation by suppressing protein kinase C (PKC) and p38 phosphorylation and inhibiting transcription factor activator protein-1 (AP-1), thereby downregulating IL-2 gene expression and accumulation (167). In addition, *P. gingivalis* may inhibit the development and proliferation of Treg cells by decreasing the secretion of TGF-β1, which may be mainly related to the type II FimA of *P. gingivalis* (34). However, this study did not compare the effects of other types of FimA at the same time, and the mechanism has not been clarified, which needs further in-depth study. Based on the above study, we found that *P. gingivalis* suppressed host adaptive immunity from the process of inhibiting antigen presentation, T cell proliferation, and activation, providing a possibility for the long-term survival of *P. gingivalis* in the host.

AS is also regulated by T-lymphocyte subsets. Among them, Th17 cells have pro-atherogenic effects and Treg cells have anti-atherogenic effects (168), thus Th17/Treg balance is closely related to AS progression. A clinical study with 1251 patients suggested that *P. gingivalis* may regulate tryptophan and kynurenine metabolism by inhibiting indoleamine 2,3 dioxygenase (IDO) activity in antigen-presenting cells, thereby promoting Th17 and inhibiting Treg proliferation, resulting in Th17/Treg imbalance and accelerating AS progression (169), but this mechanism needs to be further confirmed by *in vitro* experiments (**Figure 1**). Animal studies suggest that oral infection with *P. gingivalis* may promote Th17/Treg imbalance by increasing IL-6 expression in DCs, resulting in increasing plaque size and decreasing stability in AS (170). However, the role of *P. gingivalis* with other T cell subsets (e.g. γδ T cells, naive T cells, ApoB^+^ T cells) as well as Th cell subsets (e.g. Th9 and Th22) is unknown and needs to be further investigated in depth (12, 168).

In summary, *P. gingivalis* evades host immune cell killing through various pathways, survives in the host for a long time, induces a prolonged state of low inflammation (171), and provides an opportunity to promote the progression of AS. Although the molecular mechanism regarding the selective inhibition of immune elimination by *P. gingivalis* without suppressing the inflammatory response remains unclear, this phenomenon suggests a potentially significant threat following *P. gingivalis* evasion of immune killing. Therefore, targeting the inhibition or enhancement of one of the key links may provide a new strategy to enhance the body’s immunity and combat AS.

### 4.2 Blood circulation

**Figure 2:**
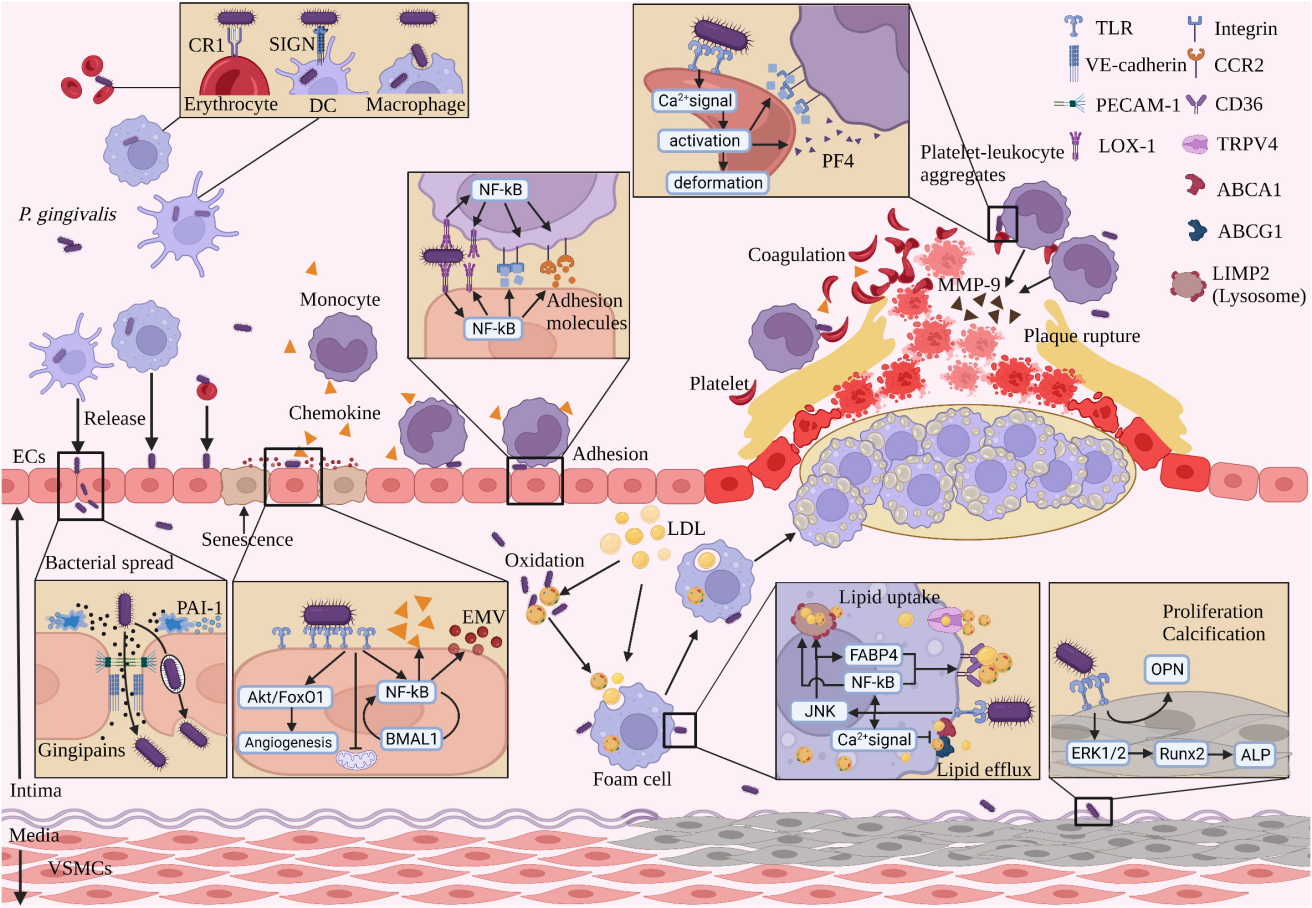
The process of *P. gingivalis* promoting AS with blood circulation. (1) *P. gingivalis* spreads to the vascular endothelium mainly in four forms with blood circulation. (2) *P. gingivalis* spreads by secreting gingipains breaking intercellular junction protein, being captured and released by ECs. (3) *P. gingivalis* stimulates ECs to produce chemokines and EMV*_pg_*, which recruit monocytes and induce senescence in adjacent ECs, respectively. (4) *P. gingivalis* mediates monocyte adhesion to the vascular endothelium. (5) *P. gingivalis* promotes lipid uptake and inhibits lipid efflux from subendothelial macrophages. (6) *P. gingivalis* activates platelets, promotes coagulation, and induces adhesion with leukocytes to form platelet-leukocyte aggregates. (7) *P. gingivalis* induces proliferation and calcification of VSMCs. (8) *P. gingivalis* induces monocytes to secrete MMP-9, promoting AS plaque rupture. (Created with BioRender.com).

*P. gingivalis* may enter the blood circulation locally from the oral cavity and then undergo systemic dissemination. Notably, *P. gingivalis* circulating with the blood may colonize the arterial endothelium, induce endothelial damage, recruit and activate platelets, promote foam cell formation and vessel wall calcification, and eventually lead to plaque rupture (172, 173) (**Figure 2**).

#### 4.2.1 Pathways of *P. gingivalis* into the blood circulation

The pathways of *P. gingivalis* into the blood circulation include three main parts, which are oral physical injury, disruption of interepithelial cell junctions by virulence factors, and immune escape.

Oral mucosal damage caused by physical stimuli such as daily activities (brushing, flossing) and oral treatments (periodontal therapy, tooth extraction) can lead to bacteremia (174). Inflammatory periodontal tissue swelling and bleeding states may also lead to the infiltration of oral bacteria into the bloodstream (175). *P. gingivalis’* various virulence factors play an important role in disrupting gingival epithelial junctional structures. *P. gingivalis* gingipains degrade E-cadherin, JAM1, and ocludin, which maintain gingival epithelial integrity and barrier function (68, 176, 177). *P. gingivalis* LPS reduces E-cadherin expression in the gingival epithelium by inducing the production of TNF-α and ROS production reduces E-cadherin expression in the gingival epithelium (178). *P. gingivalis* and its LPS may lead to a dramatic decrease in the expression of endogenous grainyhead-like 2 (GRHL2), an epithelial-specific transcription factor that regulates the expression of connexins (179). The *P. gingivalis* that enters the bloodstream also needs to evade the killing effect of various host immune cells and immune responses before it can successfully survive and multiply in the host, triggering a persistent host inflammatory response.

Systemic dissemination can occur after *P. gingivalis* has successfully entered the bloodstream and evaded host immune killing. There are four ways for *P. gingivalis* to disseminate with the blood circulation: first, it is planktonic; second, it binds to the CR1 immune adhesion receptor on the erythrocyte membrane (180); third, it binds to the SIGN receptor on DCs and enters the DCs and survives intracellularly (181); fourth, it survives in macrophages and can enter and exit the cells repeatedly (143). *P. gingivalis* in the oral cavity can follow the above four ways to reach the AS lesion site (**Figure 2**).

#### 4.2.2 *P. gingivalis* induces vascular endothelial dysfunction

*P. gingivalis*-induced dysfunction of vascular ECs is the first step in promoting AS (182, 183). Activation and dysfunction of ECs occur in AS-prone areas when stimulated by abnormal lipids, pro-inflammatory mediators, promoting secretion of pro-adhesive cytokines (184), which in turn leads to AS. Endothelial dysfunction includes changes in tissue function of contraction, spreading, barrier (185) and phenotypic changes in ECs (184). *P. gingivalis* and its virulence factors induce endothelial dysfunction in three main ways (**Figure 2**).

##### 4.2.2.1 *P. gingivalis* directly disrupts the connecting proteins and barrier function between ECs

*P. gingivalis* gingipains directly damage the vascular endothelium by degrading the endothelial adhesion molecules PECAM-1 and VE-cadherin (186, 187). Because PECAM-1 and VE-cadherin are essential for maintaining endothelial integrity and continuity, degradation of these proteins would lead to loss of endothelial integrity, increase permeability, and increase risk of direct contact of irritant molecules with deeper tissues of the vessel wall, triggering vascular inflammation (188, 189). *P. gingivalis* gingipains also hydrolyze Plasminogen Activator Inhibitor-1(PAI-1) produced by ECs, which in turn delays vascular endothelial wound healing (190). In the AS study, PAI-1 promoted the expression of Vitronectin (VN) in VSMCs by binding to LDL receptor-related protein 1(LRP1) (191), which in turn induced ECs migration and regulated vascular remodeling and healing (192, 193). Then, whether *P. gingivalis* inhibits vascular endothelial self-healing through hydrolysis of PAI-1 remains to be investigated.

##### 4.2.2.2 *P. gingivalis* affects the biological function of ECs

*P. gingivalis* not only enters and survives in ECs mediated by ICAM-1 but also releases from ECs and infects neighboring cells (194, 195). Repeated stimulation with *P. gingivalis* increases the expression of pro-inflammatory molecules (IL-6, MCP-1, GM-CSF) and vasoconstrictor molecules (Ang II) in ECs, increases monocyte adhesion to the endothelium and inhibits endothelial diastolic function (67). Similarly, a systemic Th1-type immune response occurred in mice immunized with *P. gingivalis* antigen, increasing the sensitivity of ECs to AngII and exacerbating the inhibition of endothelial diastolic function (196). However, the immunization method using intraperitoneal injection of *P. gingivalis* in this study failed to realistically mimic the natural infection state, and the mechanism of action of the Th1-type immune response and vascular endothelial sensitivity has not been fully clarified. *P. gingivalis* reduces NO production and inhibits endothelial diastolic function by inhibiting activation of the GSK-3β/BH4/eNOS/Nrf2 pathway in ECs (197). In addition, *P. gingivalis-*induced ERS occurring in ECs could both promote apoptosis and also induce an autophagic response to inhibit apoptosis (198, 199), and this paradoxical phenomenon may be related to the potential mechanism of *P. gingivalis-*promoting AS, but the further in-depth study is still needed.

*P. gingivalis* regulates the cytosolic molecules and intracellular pathways of ECs. In terms of cytosolic molecules, *P. gingivalis* GroEL upregulates TLR-4 expression on the cytosolic membrane of ECs, leading to hypersensitivity of ECs to *P. gingivalis* GroEL (50), promoting the expression of adhesion molecules (ICAM-1, VCAM-1), inducing monocyte adhesion and infiltration, and promoting AS progression (200). However, it has also been shown that TLR4 plays a protective role in AS progression (201), but the actual role of TLR4 still needs to be elucidated in the highly variable and dynamic inflammatory environment induced by *P. gingivalis*. Deeper studies revealed that *P. gingivalis*-induced inflammatory responses almost disappeared when targeted to inhibit the adapter MAL or TRAM of the Toll/IL-1 receptor (TIR) domain on ECs (202). In terms of intracellular pathways, *P. gingivalis* inhibits ECs proliferation, and promotes endothelial-mesenchymal transitions and apoptosis in a TLR-NF-κB axis dependent manner, compromising endothelial integrity and leading to the loss of ECs’ ability to repair themselves (203). Endothelial-mesenchymal transition means that activated ECs can be transformed into ectopic cell types, such as ECs into fibroblasts and calcified cells, promoting AS progression (204, 205). *P. gingivalis* LPS induces the secretion of multiple pro-inflammatory factors by macrophages in the vessel wall. These pro-inflammatory factors promote endothelial-mesenchymal transition in ECs by activating the p38-Erk1/2-p65 signaling pathway in ECs (206). In addition, *P. gingivalis* induces persistent oxidative stress and inflammatory responses in ECs through the NF-kB-BMAL1-NF-kB signaling loop with positive feedback (207). *P. gingivalis* LPS promotes the angiogenic function of endothelial progenitor cells through the Akt/FoxO1 signaling pathway (208). The enhanced angiogenic function of endothelial progenitor cells promotes AS plaque expansion and progression, as well as increases the incidence of subsequent complications such as bleeding, rupture, and thrombosis (209). Deeper studies have shown that *P. gingivalis* promotes mitochondrial mtDNA damage, increased mtROS production, and leads to endothelial dysfunction by inducing phosphorylation and translocation of mitochondrial dynamin-related protein (Drp1) in ECs (210).

The mortality and type of death of *P. gingivalis*-induced ECs were influenced by the lipid load and inflammatory status of ECs. mortality was higher in the *P. gingivalis*-induced ox-LDL pretreatment group than in the TNF-α pretreatment group, and apoptosis occurred mainly in the ox-LDL pretreatment group, whereas necrosis occurred mainly in the TNF-α pretreatment group. It suggests a synergistic relationship between *P. gingivalis* infection and AS risk factors (dyslipidemia, systemic inflammation), which together promote endothelial injury and accelerate AS progression (211). In addition, *P. gingivalis* stimulated ECs to produce microvesicular (EMV*_Pg_*) shedding, while EMV*_Pg_* may induce the conversion of adjacent endothelium to a senescent phenotype through JNK/AKT and STAT signaling pathways, promoting endothelial injury (212). It indicates that EMV*_Pg_* has significant autocrine pro-inflammatory properties. However, current studies are very limited, and it is expected to be a new indicator of vascular inflammation.

##### 4.2.2.3 *P. gingivalis* induces migration and adhesion of immune cells to the endothelium

*P. gingivalis* LPS promotes vascular inflammation and promotes AS progression through the expression of the chemokine RANTES in ECs (213, 214), which can induce leukocyte infiltration to sites of inflammation and is positively correlated with plaque instability (215, 216). *P. gingivalis* promotes the expression of lectin-like oxidized low-density lipoprotein on the cytosol of ECs and monocytes by promoting receptor-1 (LOX-1) expression, which in turn regulates ligand expression of MCP-1, ICAM-1 and E-selectin on ECs and receptor expression of CCR2 and integrin αMβ2 on monocytes, inducing monocyte migration and adhesion to ECs (217). Similarly, *P. gingivalis* and its outer membrane vesicles promote the expression of chemotactic proteins (CXCL1, CXCL2, and CXCL8) and adhesion molecules (e.g. E-selectin) in ECs (218). In addition, *P. gingivalis* promotes the secretion of macrophage migration inhibitory factor (MIF) by ECs, while MIF binds to the CD74/CXCR4 receptor complex on ECs, increases ICAM-1 expression, and promotes monocyte-ECs adhesion (219, 220). Immune cells migrate and adhere to the endothelium and then secrete multiple inflammatory factors, inducing endothelial dysfunction and promoting AS progression.

#### 4.2.3 *P. gingivalis* induces pro-coagulant effects

When vascular ECs are activated or endothelial dysfunction is present, platelets adhere to the vessel wall and are activated, releasing large amounts of chemokines that mediate the recruitment of circulating leukocytes, platelets, and coagulation factors to the vascular endothelium, promoting local inflammation and coagulation and accelerating the progression of AS (221, 222).

*P. gingivalis* activates and aggregates platelets by increasing the intraplatelet Ca2^+^ concentration, accelerating blood clotting and thrombosis. *P. gingivalis* gingipains also have a hydrolytic effect on chemokines (RANTES, MIF) released after platelet activation, inhibiting the recruitment of immune cells, preventing the clearance of *P. gingivalis* by immune cells, and producing persistent stimulation (223). Platelets activated by *P. gingivalis* express P-selectin on their surface, which binds to the receptor P-selectin glycoprotein-1 on leukocytes, forming platelet-leukocyte aggregates that not only converge a variety of leukocytes (neutrophils, monocytes) to the site of inflammation and clear bacteria but also induce a coagulation-inflammatory series of responses (224). Further studies have indicated that *P. gingivalis* induces increased platelet-neutrophil aggregate formation, enhances platelet-neutrophil interactions, and induces the release of NETs, which in turn promotes late thrombosis in AS (225, 226). However, no studies on the effects of *P. gingivalis* on other types of leukocyte-platelet interactions have been seen.

In addition, *P. gingivalis* virulence factors promote coagulation. *P. gingivalis* LPS activates platelet GTPase Cdc42 and accelerates actin assembly, which in turn induces platelet shape change and proliferation and promotes coagulation (78). *P. gingivalis* LPS promotes platelet secretion of platelet factor 4 (PF4), which is known to recruit immune cells and promote AS (221, 227). *P. gingivalis* gingipains promote P-selectin expression in platelets, which in turn promotes the adhesion of leukocytes and platelets to the endothelium, thereby promoting AS progression (228) (**Figure 2**).

#### 4.2.4 *P. gingivalis* induces foam cell formation

Macrophages are capable of uptake and clearance of modified lipoproteins. *P. gingivalis* increases intracellular lipid accumulation by interfering with lipid metabolic processes in macrophages and promotes the conversion of macrophages into “foam cells” (229, 230). The formation and accumulation of subendothelial foam cells is a key process in the formation of AS (142). The mechanism of *P. gingivalis*-induced foam cell formation is divided into three main parts (**Figure 2**):

##### 4.2.4.1 *P. gingivalis* induces lipid modification or peroxidation

*P. gingivalis* gingipains induce lipid modification and peroxidation of LDL/VLDL through hydrolysis of ApoE and ApoB-100 and enhancement of oxidative stress pathways (231, 232), while LDL receptors in the liver do not recognize modified LDL/VLDL, resulting in circulating LDL/VLDL is not cleared and continues to accumulate, promoting elevated lipids (233). Similarly, Pep19, a peptide derived from *P. gingivalis* GroEL, can also induce LDL peroxidation (234). *P. gingivalis* can also induce oxidation of HDL, where the oxidized HDL not only loses its protective function against AS but also promotes the release of TNF-α and MMP-9 from monocytes, triggering a pro-inflammatory response (235). An increase in circulating modified or peroxidized lipids is an important prerequisite for triggering foam cells, and macrophage cells initiate repair mechanisms to increase the uptake of lipids (142).

##### 4.2.4.2 *P. gingivalis* promotes lipid uptake by macrophages

Macrophage uptake of lipids is positively correlated with the number and function of scavenger receptors (CD36/SR-B2) (236). *P. gingivalis* promotes lipid uptake through the trans-activation of the CD36 promoter *via* the ERK/NF-κB pathway, which in turn upregulates CD36 expression in macrophages (237). In addition, *P. gingivalis* released large amounts of IL-1β in the form of activated CD36/SR-B2-TLR2, which in turn promoted lipid uptake by macrophages (238). Interestingly, the study also showed that IL-1β produced by *P. gingivalis*-activated CD36/SR-B2-TLR2 promoted cell pyroptosis, while ox-LDL inhibited IL-1β production and prevented cell pyroptosis in a CD36/SR-B2-dependent manner. Thus, macrophages in the vessel wall were stimulated by multiple stimuli of *P. gingivalis* LPS, ox-LDL, and IL-1β to increase lipid uptake and promote foam cell formation, but at the same time foam cells in the vessel wall were allowed to persist because pyroptosis was inhibited (238). This study provides a comprehensive analysis of the complex mechanisms underlying foam cell formation and survival in the vessel wall, suggesting that CD36/SR-B2 plays a diverse role in *P. gingivalis*-mediated AS and may be one of the important targets for regulating AS.

It was also found that the cytosolic mechanosensitive channel TRPV4 plays a key role in mediating *P. gingivalis*-promoted lipid uptake by macrophages. Under the stimulation of *P. gingivalis* LPS, the activity of the mechanosensitive channel TRPV4 in macrophages was significantly increased, the Ca^2+^ internal channel was activated, and the uptake of ox-LDL was increased. And this process was independent of the CD36 expression level (239). It is suggested that TRPV4 is another important uptake pathway independent of the scavenger receptor.

In addition, *P. gingivalis* can also affect the expression and function of fatty acid binding proteins in macrophages. It was shown that *P. gingivalis* upregulates the expression of fatty acid binding protein 4 (FABP4) through activation of the JNK pathway and forms a positive feedback loop that promotes lipid uptake and increases intracellular lipid accumulation by macrophages (240, 241).

##### 4.2.4.3 *P. gingivalis* inhibits lipid efflux from macrophages

*P. gingivalis* LPS promotes lipid accumulation in macrophages by activating the JNK-c-Jun/AP-1 pathway to up-regulate CD36 (lipid uptake) expression, while down-regulating ATP-binding cassette transporterA1 (lipid efflux) expression by enhancing calpain activity (230). Secondly, *P. gingivalis* may inhibit cholesterol efflux by activating NF-κB and JNK signaling pathways, which in turn upregulates lysosomal integral membrane protein 2 (LIMP2) expression levels in macrophages (242). In addition, *P. gingivalis* inhibited the activity of cholesterol efflux-related enzymes (ABCG1 and CYP46A1) and promoted lipid accumulation in macrophages by enhancing Ca^2+^ signaling and promoting ROS production (243). Current studies on the inhibition of lipid efflux from macrophages by *P. gingivalis* are relatively limited. It mainly involves the functions of membrane transport proteins and receptors, and related enzymes, and its effects are attributed to the triggering and persistence of inflammatory signaling pathways.

#### 4.2.5 *P. gingivalis* induces vascular calcification

Vascular calcification is the pathological deposition of hydroxyapatite minerals in the vascular system in the vessel wall, which in turn promotes AS progression. VSMCs are the key cell type involved in vascular calcification and exhibit phenotypic conversion. Various immune cells infiltrate the lesion in the early stages of AS formation, producing pro-inflammatory factors and regulatory molecules that induce the migration of contractile phenotype VSMCs, originally located in the interstitial layer, to the intimal layer and convert to a synthetic phenotype that contributes to the deposition of hydroxyapatite minerals in the vessel wall (244). VSMCs of the synthetic phenotype in the intimal layer are the first cells to appear at the site of impending AS lesions (245).

*P. gingivalis* promotes calcium deposition in VSMCs and promotes the transdifferentiation of VSMCs to osteoblast-like cells (246). It is hypothesized that various inflammatory cytokines (TNF-α, IL-β) induced by *P. gingivalis* promote vascular calcification by upregulating the expression of osteogenic-related genes (ALP, RUNX2) in VSMCs (247, 248), but further validation is needed. In addition, different virulence factors of *P. gingivalis* also play an important role in inducing vascular calcification. *P. gingivalis* LPS stimulates VSMCs proliferation and calcification, leading to vascular calcification (249). *P. gingivalis* gingipains induce VSMCs proliferation and conversion to synthetic type, increase bone bridge protein (OPN) expression, and promote vascular calcification (250). *P. gingivalis* OMVs promote vascular calcification by activating the ERK1/2- Runx2 signaling pathway to promote calcification in VSMCs (251).

In recent years, some studies using complex cell models to simulate the complex environment *in vivo* have also emerged. *P. gingivalis* LPS promoted calcification in VSMCs co-cultured with human periodontal ligament cells. This study indicates that the calcification effect is not only derived from the stimulation of *P. gingivalis* LPS, but also the secretion of various pro-inflammatory factors by *P. gingivalis* LPS-stimulated human periodontal ligament cells (252). This study somewhat mimics the *in vivo* environment in which periodontitis and cardiovascular disease coexist. There is also one that simulates the relationship between *P. gingivalis* and the calcification of VSMCs under hyperglycemic conditions. Under hyperglycemic conditions, *P. gingivalis* enhanced smad1/5/8-runx2 signaling by activating TLR-4 and ERK1/2-p38 signaling and promoting bone morphogenetic protein 4 (BMP4) autocrine regulation, which in turn induced calcification in VSMCs (253) (**Figure 2**).

#### 4.2.6 *P. gingivalis* promotes plaque rupture

When AS is advanced, immune cells recruited by *P. gingivalis* infiltrate the vessel wall and secrete MMPs, such as MMP-1 and MMP-9, which degrade the collagen fibers of the plaque fibrous cap, leading to plaque rupture and bleeding (254).

*P. gingivalis* Mfa-1(short or minor fimbriae) induces differentiation of blood monocytes into DCs and promotes the expression of MMP-9 which in turn increases the risk of plaque rupture (255). Similarly, *P. gingivalis* LPS promoted MMP-9 expression and activity in monocytes (256). In addition, *P. gingivalis* gingipains hydrolyze the complement component C5, leading to local accumulation of C5a accumulation (114, 257). C5a is the only biologically active fragment following the action of *P. gingivalis* gingipains that induces increased expression of MMP1 and MMP9 in macrophages in plaques (114, 118), leading to degradation of the extracellular matrix and rupture of plaques (123). Studies on the promotion of AS plaque rupture by *P. gingivalis* are scarce and remain to be investigated (**Figure 2**).

### 4.3 Lymphatic circulation

The initial lymphatic vessels, the beginning segment of the lymphatic tract, lacking continuous basement membrane and perivascular wall cells, may serve as an ideal pathway for pathogen transmission through the lymphatic circulation (258–260). *P. gingivalis* may enter the submandibular and submental lymph nodes through the initial lymphatic vessels, and they may subsequently drain down the superficial/deep jugular lymph nodes to the jugular venous trunk for further metastasis to more distant organs, such as AS plaques, liver, etc (261). *P. gingivalis* that circulates with lymph may accelerate AS progression by inhibiting T cell and B cell activation and cholesterol reversal of transport function and promoting Th17/Treg imbalance in the spleen.

#### 4.3.1 *P. gingivalis* inhibits the immune function of the lymphatic system

DCs, as the cells with the highest antigen-presenting capacity, are important for the activation of T and B cells in the lymphatic system. *P. gingivalis* gingipains hydrolyze the chemokine CCL21 in periodontal tissue and inhibit the entry of DCs into the initial lymphatic vessels (262, 263), which in turn reduces the activation of T and B cells in the lymphatic system by DCs (264). At the same time, *P. gingivalis* enters and survives in DCs by binding to SIGN receptors on DCs through its fimbriae, which helps *P. gingivalis* evade immune killing by the lymphatic system and promotes AS progression (255). *P. gingivalis* that survives in DCs does not affect CCR7 expression on the cytosol, but significantly upregulates CXCR4 expression (265). CCR7 mediates the homing of DCs to secondary lymphoid organs, whereas CXCR4 mediates the migration of DCs to sites of active vascular remodeling, such as AS (266). Thus, *P. gingivalis* may “hijack” DCs and enrich intracellular *P. gingivalis*-containing DCs in inflammatory vascular sites.

*E. coli* LPS can impair the recruitment of immune cells (DCs, macrophages) associated with lymphatic vessels and reduce the contractility of lymphatic vessels, affecting the function of the lymphatic pump (267). However, whether *P. gingivalis* LPS has similar functions has not been reported and needs to be urgently investigated.

#### 4.3.2 *P. gingivalis* attenuates the cholesterol reversal function of lymphatic vessels

Lymphatic vessels can efficiently reverse cholesterol transport from multiple tissues (268), including the arterial wall, a process that is positively dependent on HDL uptake and transport by scavenger receptor class B type I (SR-BI) on lymphatic ECs (269). The density of capillary lymphatic vessels present in AS lesions increase with plaque progression (270), and proliferating capillary lymphatic vessels may inhibit AS progression by reversing cholesterol transport (271). *P. gingivalis* may generate large amounts of ROS by interfering with the function of the mitochondrial respiratory chain, leading to endoplasmic reticulum stress (ERS). Subsequently, it triggers an unfolded protein response (UPR), which promotes CHOP gene expression and suppresses SR-BI expression (272–274). This in turn inhibits the process of cholesterol reversal in the arterial wall and accelerates the progression of AS. However, there is a lack of direct studies on the inhibition of SR-BI expression on lymphoid ECs by *P. gingivalis*.

SR-BI is present in the liver, macrophages, and ECs. In addition to its function of uptake and transport of HDL, SR-BI also has an induced anti-inflammatory effect (275). For example, SR-BI binding to HDL inhibits the NF-κB pathway, decreases the inflammatory response of macrophages to LPS, and significantly reduces the secretion of various pro-inflammatory factors (276). However, the role of SR-BI in *P. gingivalis* promoting AS and its mechanism need further study.

#### 4.3.3 *P. gingivalis* induces Th17/Treg imbalance in the spleen

The spleen is a peripheral lymphoid organ dominated by T cells and B cells, and is the center of cellular and humoral immunity in the body. (277). Among them, Th17 promotes AS progression, while Treg inhibits AS progression. Therefore, Th17/Treg balance can control inflammation and may play an important role in plaque stability (278).

The DNA genome of *P. gingivalis* can be found in the spleen of Apoe^shl^ mice orally infected with *P. gingivalis* (47), and *P. gingivalis* promotes Th17 proliferation and differentiation by upregulating the expression of IL-6 in DCs in the spleen, leading to Th17/Treg imbalance, increasing plaque area and decreasing plaque stability (170). Intravenous administration of *P. gingivalis* to ApoE^-/-^ mice showed a significant increase in Th17 and Th17-related molecules in the spleen and heart, as well as a significant increase in AS plaque area (279). In addition, *P. gingivalis* LPS did not induce the expression of NK cell CD69 in the spleen (280), which in turn promoted Th17 differentiation and inhibited Treg differentiation, leading to Th17/Treg imbalance (281), but the mechanism needs to be further investigated.

## 5 Conclusion and outlook

In recent years, the relationship between *P. gingivalis* and AS has received increasing attention. *P. gingivalis* can occur immune escape, promote local inflammation and plaque formation in blood vessels, inhibit immune defense and cholesterol transport function of the lymphatic system, and promote the progression of AS. This paper summarizes the recent research results related to the promotion of AS by *P. gingivalis* through immune response and provides new insights to further reveal the potential mechanisms and associations between *P. gingivalis* and AS.

In addition, this paper shows the different cell subpopulations associated with AS discovered by single-cell sequencing technology in recent years, which updates the understanding of the nature and function of cell subpopulations in AS. It provides the conditions to precisely grasp the cellular level of AS pathogenesis. However, unfortunately, single-cell sequencing studies on *P. gingivalis* and AS have not been reported yet. It is believed that shortly, the use of single-cell sequencing technology will certainly provide important theoretical support for the prevention and treatment of *P. gingivalis* to accelerate the progression of AS.

## Author contributions

QR and PG wrote the manuscript. WQ, JL, MX, LX and SZ made the figures and edited the manuscript. DM, and JN administrated and supervised the whole research project. All authors have read and agreed to the published version of the manuscript. All authors contributed to the article and approved the submitted version.
